# Potential of Flax Shives and Beech Wood-Derived Biochar in Methylene Blue and Carbamazepine Removal from Aqueous Solutions

**DOI:** 10.3390/ma15082824

**Published:** 2022-04-12

**Authors:** Hicham Zeghioud, Lydia Fryda, Angélique Mahieu, Rian Visser, Abdoulaye Kane

**Affiliations:** 1 UniLaSalle-Ecole des Métiers de l’Environnement, Cyclann, Campus de Ker Lann, 35170 Bruz, France; lydia.fryda@unilasalle.fr (L.F.); angelique.mahieu@unilasalle.fr (A.M.); abdoulaye.kane@unilasalle.fr (A.K.); 2 Department of Energy Transition, Dutch Institute of Applied Research TNO, Westerduinweg 3, 1755 LE Petten, The Netherlands; rian.visser@tno.nl

**Keywords:** adsorption, pollutant, biochar, flax shives, beech wood, activation, surface oxidation

## Abstract

Flax shives and beech wood residues represent biomass streams that are abundant in Northwest Europe. These primary feedstocks were evaluated for their suitability to produce biochar as a low environmental-impact adsorbent. The efficacy of the produced biochars was tested by their adsorption capacity towards methylene blue (MB). A series of adsorption tests with carbamazepine is also presented, focusing on the better performing beech wood biochar. Post treatment of the biochars with citric acid (CA) and oxidation of the surface by heating at 250 °C in a muffle oven were carried out to enhance the adsorption capacities of both flax shives biochar (FSBC) and beech biochar (BBC). The resulting physicochemical characteristics are described. The thermally treated biochars have specific surface areas of 388 m^2^·g^−1^ and 272 m^2^·g^−1^ compared to the untreated biochars with 368 and 142 m^2^·g^−1^ for BBC and FSBC, respectively. CA treatment leads to enhancement of the oxygenated surface functional groups and the adsorption capacities of both studied biochars. The non-linear Langmuir and Freundlich models show the best fit for both the isotherm data for MB and the CMZ adsorption with a good correlation between the experimental and calculated adsorption capacities. The effect of adsorbent dosages and initial concentrations of MB and CMZ on the adsorption efficiency is discussed. It can be concluded that beech biochar is a very promising pollutant adsorbent only requiring a mild, low-cost, and low-environmental impact activation treatment for best performance.

## 1. Statement of Novelty

Significant quantities of biomass residues exist in Europe, currently valorized mainly for heat production. We propose a more optimal valorization path for those residues by converting them to biochar with bioenergy as co-products. The creation of such a value chain will reduce the dependency on imported and often fossil-based active carbon and will contribute to CO_2_ emission reduction and regional development. This study suggests low environmental impact and economically attractive methods to improve the quality of biochars by functionalizing and activating the materials produced in a standard medium-temperature pyrolysis process. The results identify beech wood biochar as a promising low-cost and low-impact material for environmental applications in specific water treatment. This work is the result of a collaboration among researchers in the field of novel process development, water treatment, and material science, aiming to evaluate the specific requirements of biochar that need to conform to certain quality standards and, in this study, in water treatment. Consequently, this manuscript is of interest to researchers in the fields of biochar production process optimalisation, effluent treatment, characterization of materials, and optimization of biochar functionality, and is presented in an easy-to-follow language.

## 2. Introduction and State of the Art

There are numerous physical, chemical, and biological methods to treat and purify wastewater, each showing specialized aspects over others [1,2]. The utilization of activated carbon to remove pollutants from air or water streams in municipal and industrial processes such as wastewater treatment plants, groundwater remediation, and air purification or oil removal, is an effective separation technique in terms of simplicity of design, reliability and efficiency of pollutant removal, and ease of operation, and is insensitive to toxic substances [3]. In general, however, it is an expensive step in the cleaning process. The global active carbon market size was estimated at USD 4.72 billion in 2018 [4] and is expected to expand further in the coming period. Increasing demand for water treatment and sewage treatment applications is expected to be a key growth driver.

Active carbon is prepared by pyrolysis of carbon-rich feedstock including coal, peat, nutshell, coconut husk, wood, and municipal solid waste, at a high temperature and is further activated to increase the adsorption capacity of the carbon material. The process of active carbon production is energy- and material-consuming and if fossil feedstocks or materials that have been transported over a long distance are used the environmental footprint can be considerable [3,5].

The adsorption capacity of active carbon (AC) for various contaminants is linked to its very large surface area and porosity, as well as to its surface physicochemical properties [6]. The surface chemistry of active carbon is the result of the oxygen functional groups that populate the pores on activated carbon’s internal and external surfaces: carboxyl, lactones, phenols, and carbonyls. These surface functional groups, in combination with the accessible porosity and the surface area, bind pollutants following various mechanisms (pore diffusion, electrostatic bonds, π–π interactions, chemical bonds) [7,8]. The result of these processes is a material with enriched oxygen-containing surface functional groups and a superior porosity compared to that of its parent material.

During the last decade, several low-cost adsorbents based on agro-residues have been used for the removal of organic and inorganic pollutants from aqueous solutions [9,10]. Examples of such materials are rice husks, sugarcane bagasse, eucalyptus bark sawdust [9], pine needles, prawn shell activated carbon, and fruit kernel powder [10].

Biochar produced from local, often under valorized residual biomass is gaining attention nowadays as a sustainable platform carbon material [11] and an alternative to active carbon. Local agro-residues offer an abundant source of feedstock for biochars, minimizing long transport times and potentially being cheaper than active carbon. Moderate temperature gasification or pyrolysis offers the advantage of co-producing a fuel gas together with biochar of suitable quality for agronomic applications [12,13]. These biochars have been explored as a peat replacement in soilless substrates in horticulture (Interreg Project HortiBlue-C, https://www.horti-bluec.eu/en, accessed on 28 March 2022) and are currently being evaluated as active carbon substitutes or partial replacements for waste or stormwater treatment applications (Interreg Project THREEC, https://threec.eu/, accessed on 28 March 2022).

Beech wood chips are an abundant woody feedstock of high quality and are low in ash. For the current trials, beech wood pellets from Germany were used (Rettenmaier Räucher Gold HBK). This beech wood is of a constant composition and particle size distribution (2.5 to 3.5 mm) and is therefore suitable for benchmarking and standardizing the test protocol. Besides, beech wood residues are also abundant in Northwest Europe as a residue from the maintenance of parks and municipal forests.

Flax shives were collected from northwest France. Flax is grown for oil or fiber, depending on the variety. Flax shives are a by-product obtained from the scutching process that turns straw flax into finer fibers. Flax shives are the woody, nonfibrous part of the stems (not including the seeds) that remain after the extraction of the bast fiber (the fiber surrounding the phloem in the stem) in flax fiber production.

During the biomass pyrolysis process into biochar, aromatization reactions take place that lead to the formation of fused ring aromatic structures and aromatic C–O groups [14]. Research on steering biochar properties through controlling pyrolysis temperature and residence time suggests that these operating parameters mainly influence the development of biochar surface area and porosity, though it is acknowledged that it is the functional groups on biochars surfaces that are strongly correlated with the high adsorption capacities of chars towards certain compounds [15]. Further modifying biochar after pyrolysis using a subsequent activation step would be an effective method for grafting functional groups on the biochar surface that are already formed during pyrolysis at lower temperatures, but diminish at high pyrolysis temperatures [10].

Activation is the process of treating a char or biochar either physically, by heating the material under steam, CO_2_, or limited oxygen at high temperatures, or chemically, by treating the material in an acidic or basic environment in order to enrich the surface with a finer porosity, active sites, and surface functional groups [16,17]. Chemical activation involves strong mineral bases and acids to alter the physiochemical surface properties by introducing chemical entities on the surface of the biochars creating specific sites on the surface necessary for the adsorption of targeted compounds. Thus, chemical activation may have an environmental impact due to the use of chemicals [18,19,20]. Physical activation is generally energy- and cost-intensive because of the very high temperatures required. Considering biochar as a sustainable alternative to active carbon, the activation process following the production must also consider lower-impact alternatives. The use of weak organic acids to graft specific functional groups is suggested [6,8], such as carboxylic acid (–COOH) groups on biochar surfaces.

Weak organic acid application and subsequent disposal during biochar functionalization is less hazardous and does not cause corrosion [19,20]. Carboxylic acids such as acetic acid (CH_3_COOH), citric acid (C_6_H_8_O_7_), tartaric acid (C_4_H_6_O_6_), oxalic acid (C_2_H_2_O_4_), and malic acid (C_4_H_6_O_5_) contain the carboxyl functional group –COOH on the biochar surface through esterification which binds with pollutants in aqueous solutions. Organic acids have been successfully used as modification agents for biochar activation [18,20,21], especially citric acid which is used to modify various waste biomass materials for the adsorption of heavy metal ions and dyes. Controlling the surface chemical composition of biochars through functionalization offers high added-value applications to low-cost, or negative-cost biomass residues. It has been shown that some biochar materials, despite a low surface area, can have very high adsorption capacities due to their high surface functionality [22,23]. Therefore, the application of organic acids for the functionalization of biochars can be an economically viable and environmentally friendly method to enhance the adsorption capacity of biochar [19]. However, research is not yet conclusive regarding the effect of surface functionality on the adsorption capacity of specific organic and inorganic compounds in water.

In addition, simply oxidizing the biochars at low temperature is another low-impact method to add oxygen groups on the surface [24]. This mild activation process is likely to increase the porosity and surface functionality to a lesser degree compared to commercially available active carbon.

Methylene blue is a cationic dye found in wastewater, widely used for the coloring of textiles [25]. Methylene blue has been widely studied because of its known strong adsorption onto solids, and it often serves as a model compound for removing organic contaminants and colorants from aqueous solutions.

Carbamazepine (CMZ) is one of the most-used pharmaceuticals worldwide. It is widely used to treat epileptic seizures and nerve pain. It does not easily attach to inorganic materials so it can stably remain in the aqueous phase, and therefore represents a potential risk to the environment and human health. It is well-known as a refractory molecule to traditional water and wastewater treatment processes and particularly to biological processes. Biochar is a sustainable adsorbent for the removal of recalcitrant contaminants such as CMZ. Alternative adsorbents are activated carbon, silica-based materials, chitosan-based materials, granular carbon nanotubes/alumina hybrids, and zeolites [26].

The available literature is rich in the identification of all underlying mechanisms of pollutant adsorption on biochar and activated biochar surfaces. The adsorption process is multifactorial and affected by carbon characteristics, pollutant characteristics, specific area, porosity, polarity, hydrophilicity, wastewater characteristics (concentrations of biologic organic matter and specific organic micropollutants), and molecular interactions. The physicochemical properties of biochar need to be correlated with the adsorption mechanisms to remove organic and inorganic pollutants (electrostatic interaction, ion exchange, pore filling, and precipitation) to provide a systematic understanding of the adsorption mechanisms for each pollutant under given adsorption parameters [27]. The mechanism and processes explaining the formation of the various functional groups on the surface of chars during carbonization/pyrolysis are the objectives of the current research.

The objective of this research is to evaluate whether the suggested low environmental impact and low energy-consuming functionalization of biochars is an effective, meaningful way to enhance the adsorption capacity of biochars towards pollutants commonly found in waters such as MB and CMZ, in order to set the frame for follow-up research combining the knowledge of mild functionalization [6,8] and the adsorption of pharmaceuticals and colorants. There is a broad variety of biochars based on feedstock and production conditions, as well as a broad range of activation and functionalization processes. The present study does not suggest an inventory of biochars and functionalization methods toward well-performing molecules, as this is an ongoing task within national and EU projects and addresses the broad problem of the standardization of biochars. The European Biochar Certificate (EBC) (https://www.european-biochar.org/en, accessed on 28 March 2022) is well-known for its efforts in this area, and already suggests biochar qualities for certain applications. However, there is still a long way to go before one can rely on databases that will suggest biochars, their activation/functionalization methods, and the best applications for them. This work contributes to the ongoing research on low environmental impact and economically attractive methods to improve the adsorption performance of biochars by functionalizing and activating the materials produced in a standard medium-temperature pyrolysis process.

The current research results discuss the suitability of biochars from flax shives and beech wood as pollutant adsorbents. The biochars were post treated with either a mild organic acid (citric acid), or with oxidation of the surface by heating up briefly at 250 °C in a standard muffle furnace. The materials were subjected to (i) physical and chemical characterizations and (ii) batch adsorption tests to evaluate the adsorption capacity for methylene blue dye and carbamazepine in aqueous solutions. Since this study focuses on evaluating and optimizing certain biochars, it is necessary to rely on well-documented references on the adsorption behavior of these molecules to draw sound conclusions by comparing them with data in the literature. MB was chosen in this study because of its known strong adsorption onto carbon surfaces and its recognized effectiveness in characterizing adsorptive material and is being used as an indicator of the adsorption capacity of carbon materials, together with the iodine number.

## 3. Materials and Methods

### 3.1. Biomass Feedstock and Biochar Production

The ultimate and proximate analysis of the feedstock is shown in Table 1, data are property of TNO (Phyllis database, https://phyllis.nl/, accessed on 28 March 2022, and HortiBlue-C project). These two feedstocks do not show any large differences in their composition, they are not contaminated, and the ash content is relatively low; therefore, there are no contraindications as to their conversion into biochar based on EBC suggestions. The high calcium content of the flax shives is expected to influence the pH of the resulting biochar, as will be discussed in the relevant section on the biochar characterization results.

**Figure 1:**
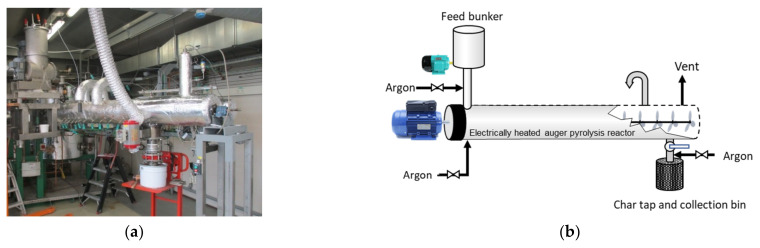
(**a**) Full view of the auger-fed pyrolysis reactor and (**b**) schema of the counter-current flow.

The pyrolysis trials have been performed at TNO (Dutch Institute of applied research) in the Netherlands, in an indirectly heated auger-fed (screw) reactor (Figure 1) [11]. The reactor is a cylindrical horizontal vessel, which rotates slowly around its axis. The turning screw is carrying the material through the reactor and secures sufficient residence time. Steam was used for treating the biochar, while inert gas was used to avoid tar condensation during the harvest of the char. The pyrolysis unit is dimensioned to convert typically 3–5 kg/h of feedstock in an O_2_-free atmosphere at temperatures up to 650 °C. The feedstock residence time was adjusted to 15 min. The produced biochar was preserved in airtight metal containers.

### 3.2. Post Treatment of the Biochars

The biochars were produced at a pyrolysis temperature of 650 °C and steam was added at the same temperature. Under such conditions, some activation already takes place by revealing porosity and creating more surface area compared to a biochar produced at lower temperature without the addition of steam.

#### 3.2.1. Citric Acid Treatment (CA)

The biochars were soaked in citric acid solution of 1 M in a 1/10 mass ratio (1 g biochar in 10 mL acid). After 24 h the paste was first dried at 50 °C to evaporate the excess moisture and then introduced into an oven, heated up to 175 °C for 2 h and then quenched in water. The biochars were rinsed until clean. Then the materials were left to dry overnight at 80 °C, ground to powder, and stored in a dry container at room temperature.

**Figure 2:**
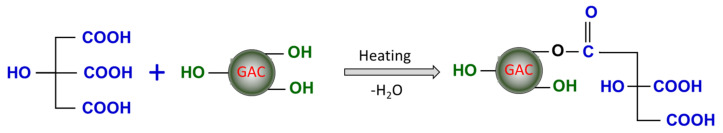
Citric acid modification of granular activated carbon (GAC).

According to Rashid et al. [28], when heated, CA dehydrates to yield a reactive anhydride which reacts with the hydroxyl groups present on the biochar surface to form carboxylated BC shown in Figure 2 for the case of active carbon.

CA was probably already dehydrated and formed carboxylated BC so heating further did not affect the quality of the product.

#### 3.2.2. Low-Temperature Surface Oxidation (Oxide)

Biochar was ground and placed in an air oven for 1 h at 250 °C. The biochars were left to cool down to 105 °C before being stored in a dry place until use.

The biochars tested and presented in this work are shown in Table 2. The only water-washed biochar signifies materials that were only rinsed under demineralized water and dried to safeguard a clean material during the adsorption tests. All materials were washed before use; therefore, this treatment can be considered a reference common to all.

### 3.3. Adsorbate

Methylene blue (C_16_H_18_N_3_SCl.3H_2_O) is a cationic dye with a net-positive charge, highly soluble in water (log K_ow_ = 0.75 or 43.6 × 10^3^ mg/L at 25 °C), pKa = 3.8, and molecular projected area of 130 Å^2^ [18]. Methylene blue was obtained from Merck and was used without further purification. The solution was prepared by dissolving the required amount of dye in distilled water. MB has a molecular weight of 319.85 g·mol^−1^, which corresponds to the heterocyclic aromatic chemical compound with the molecular formula C_16_H_18_N_3_SCl. Carbamazepine was purchased from Sigma Aldrich in pure and dry form. The chemical formula of CMZ is C_15_H_12_N_2_O, its MW (g·mol^−1^) is 236.27. CMZ is at nonionic state at the typical pH values of water and its log K_ow_ = 2.45 indicates a lower solubility than MB.

**Figure 3:**
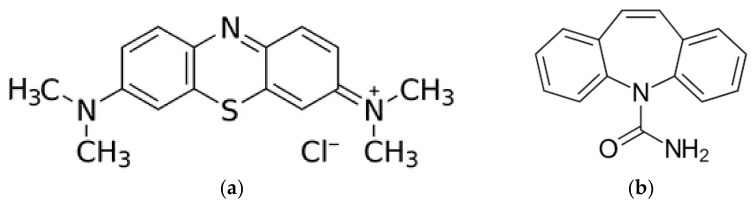
The chemical structure of (**a**) methylene blue chloride salt and (**b**) carbamazepine.

The structure of both Methylene blue dye and carbamazepine are shown in Figure 3.

Stock solutions were prepared by dissolving an accurately weighed 100 ± 0.005 mg of MB dye in 1 L of distilled water. Various concentrations via dilution process (5–100 mg/L) of MB dye solution were prepared by diluting the stock solution. The solutions were covered with aluminium foil and stored in a dark place to prevent UV degradation. The Carbamazepine solutions were prepared by dissolving a quantity of CMZ in ethanol and adding distilled water to form the various concentrations. A Shimadzu UV-visible spectrophotometer (SHIMADZU UV-1800, Canby, OR, USA) was used for the quantification of the concentrations at λ_max_ 664 nm for methylene blue and λ_max_ 265 for carbamazepine. The calibration of the UV-vis spectrophotometer was carried out by plotting various optical densities at various concentrations of methylene blue dye in distilled water. The absorbance and concentration are plotted (Figure 3a,b) to obtain the calibration data for the two components. The calibration curves are available in the Supplementary Materials (Figure S1).

### 3.4. Characterization of Biochars

The ash content was established gravimetrically after submitting the biochar to a calcination process under an air atmosphere at 550 °C for 6 h. For the surface functional groups of biochars, Fourier transform infrared spectroscopy (FTIR) was employed using a spectrometer from ThermoFisher (Waltham, MA, USA). Char samples were mixed with KBr at a ratio of 1: 180 and the wavenumber range of infrared scanning was 400–4000 cm^−1^. The morphology of the biochar surfaces was determined using scanning electron microscopy (SEM) from Jeol (Akishima, Japan). The specific surface area was determined by N_2_ adsorption at 77 K via Brunauer–Emmett–Teller (BET) (Quantachrome Instrument, Boynton Beach, FL, USA). The concentration of heavy metals (HMs) was measured by an inductively coupled plasma optical emission spectrometer (ICP-OES) (Varian, Palo Alto, CA, USA). Prior to the analysis, the samples were degassed at 200 °C for 9 h. Thermogravimetric (TGA) analysis of biochar before and after modification was carried out using Q600 STD Thermogravimetric Analyzer (TA Instruments, New-Castle, DE, USA) starting from room temperature to 800 °C with heating rate of 10 °C/min and under nitrogen atmosphere.

The pH of the solution is a parameter that affects the capacity of different adsorbents for the uptake of pollutants. The interactions between adsorbent surface functionalities and the either negatively or positively charged pollutant molecules may influence their uptake [29]. The pH of the solution can affect the adsorption behavior when ions are present in the solution, as in the case of the cationic dye methylene blue.

Biochar pH was measured in 1:10 biochar: water (deionised water; DIW) ratio after 24 h shaking at 25 °C. After this, samples were allowed to stand for 30 min and then pH was measured using a pH system. Another parameter that influences the adsorption capacity specifically of ionic molecules is the pH at the point of zero charge. The combined influence of all the functional groups of activated carbon determines pHpzc, i.e., the pH at which the net surface charge on carbon was zero. At pH < pHpzc, the carbon surface has a net positive charge, whereas at pH > pHpzc the surface has a net negative charge [30].

The pH at the point of zero charge (pH_pzc_) of the biochars was evaluated using a titration procedure followed by Salazar Rabajo et al. and Leyva-Ramos [31,32] and briefly described here. Six neutralizing solutions of pH between 2 and 12 were prepared by adding NaOH or HCl solutions of either 0.1 M or 0.01 M to a 0.01 M NaCl solution. A mass of 0.15 g of biochar and 50 mL of neutralizing solution were added into a 100 mL Erlenmeyer glass, and nitrogen gas was bubbled for 5 min to prevent the formation of carbonates in the solution. The containers were covered and the solution was agitated at 300 rpm for 24 h. The above procedure was repeated without adding biochar; these experiments were designated as blanks. After 24 h, the final pH of the solutions was measured and the potentiometric curves corresponding to solutions with biochar and without biochar were plotted. The intersection of both curves was the pH_pzc_ of biochar.

### 3.5. Experimental Protocol of Batch Adsorption

The batch adsorption experiments were conducted in a set of 250 mL Erlenmeyer flasks containing the adsorbent (biochar) and 100 mL of either MB or CMZ solution in demineralized water at various initial concentrations of *C*_0_. Variable quantities of biochar were added to the flasks, from 0.05–0.5 g per flask. The flasks were agitated in an isothermal water-bath shaker at 130 rpm and 20 ± 1 °C for 24 h. After filtration through a 0.21-micron syringe-supported nylon filter, the equilibrium concentrations in the solution were quantified using a Shimadzu UV-visible spectrophotometer. The pH of the solution was monitored, with initial values of the biochar pollutant solution being about 6.5.

The dye uptake by the biochar *q_e_* (mg/g) and the percentage dye removal are determined applying Equations (1) and (2).

(1) $$q_{e}=\frac{V}{m}\left(C_{0}-C_{e}\right)$$

(2) $$\mathrm{Removal}\;\mathrm{efficiency}=\frac{\left(C_{0}-C_{e}\right)}{C_{0}}\times 100\%$$

where *q_e_* is the amount of adsorbate in mg per gram of adsorbent, *C_0_* and *C_e_* are initial concentration and equilibrium concentration, respectively, of the pollutant (mg/L) as calculated by the calibrated spectrophotometer absorbance signal, *V* is the volume of the solution (L), m mass of adsorbent (g). From the data on *C_e_*, *q_e_*, and *C*_0_ the saturation curves and the isotherms were drawn. Further testing with CMZ was carried out only with the biochar that showed better adsorption efficiency on methylene blue.

To gain insight into the role of the pH in the adsorption, some supplementary tests were carried out with varying pH values for the two model pollutants. In aqueous phase adsorption systems including active carbons and clay materials, factors that affect the adsorption of dyes onto biochar and its modified forms are solution pH, temperature, initial dye concentration, or the presence of other substances [32]. Variation in the solution pH may result in a change in the surface charge of the sorbent and the degree of ionization of the absorbing ion. For example, if the surface of the adsorbent is positive in an acidic pH range, its sorption capacity will be low for positively charged species. The surface of the solid becomes repulsive to the contaminant and thus leads to reduced uptake by the adsorbent and its modified forms in acidic solutions. In the case of a cationic pollutant, uptake is higher owing to the negative charge of the substrate surface under the entire range of pH studied.

### 3.6. Adsorption Isotherm Modelling

#### 3.6.1. Langmuir Fit

To identify the distribution of adsorbate molecules on the biochar surface, equilibrium isotherm modeling is used to study the dye–biochar adsorption mechanism. In this work, the sorption mechanisms of carbamazepine and a cationic dye onto various biochars were assessed by employing the two most well-known isotherm models (Freundlich, Langmuir).

The Langmuir sorption isotherm is applied to equilibrium assuming monolayer adsorption onto a surface with a finite number of identical sites, suggesting that all active binding sites are energetically the same and adsorb only one dye molecule.

The non-linear Langmuir equation takes the following form [33]:

(3) $$q_{e}=\frac{q_{m}K_{L}C_{e}}{1+K_{L}C_{e}}$$

where, *q_m_* (mg/g) is the single layer uptake capacity (maximum) of the biochar; *K_L_* (L/mg) is the affinity constants for the Langmuir model.

#### 3.6.2. Freundlich Isotherm

The Freundlich equation for heterogeneous surface energy systems assumes a multilayer adsorption process and uneven energy distribution of active sorption sites [34,35]. The Freundlich model represents sorbent surface heterogeneity. The non-linear mathematical form of Freundlich model is expressed as follows:

(4) qe=kfce1n

where *K_f_* and 1/*n* are Freundlich constants. The parameters *K_f_* and 1/*n* are related to sorption capacity and the sorption intensity of the system.

## 4. Results and Discussion

### 4.1. Surface Morphology Results: Scanning Electron Microscopy (SEM)

**Figure 4:**
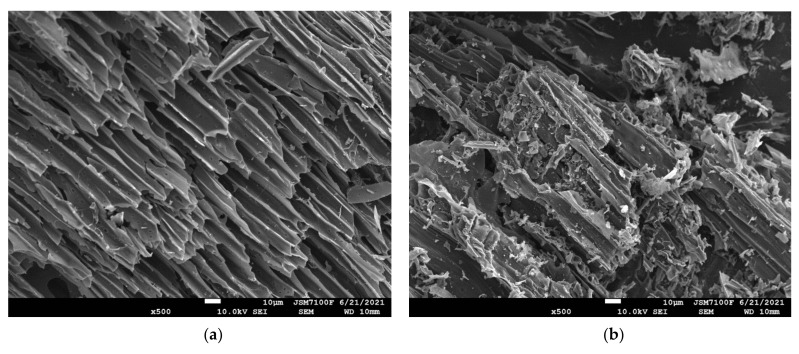

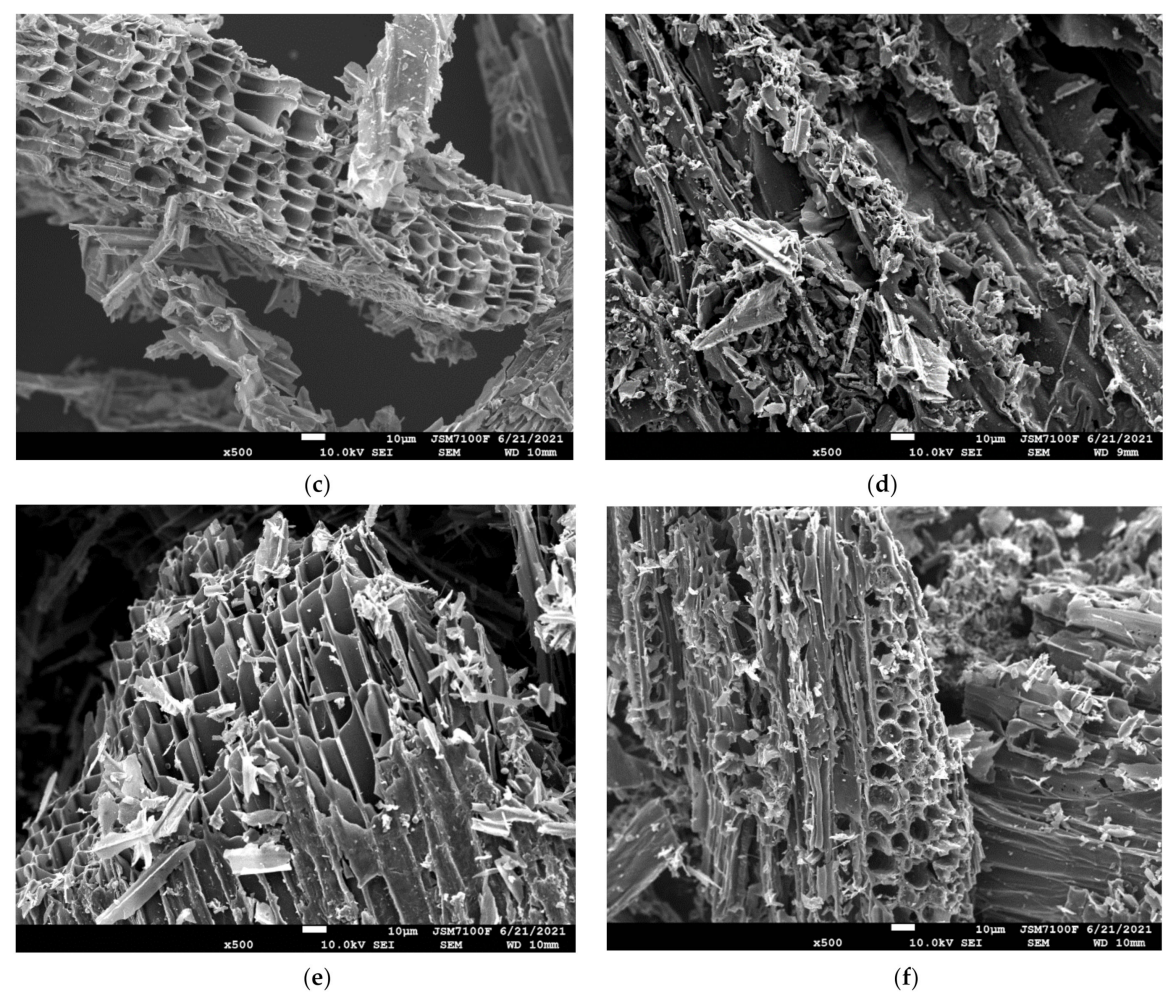
SEM image of flax and beech biochars at untreated, citric acid-treated (CA), and thermally treated forms, magnification: 500×. (**a**) Flax shives biochar non-treated, (**b**) Beech wood biochar non-treated, (**c**) Flax_shives biochar Citric Acid treated, (**d**) Beech_wood biochar Citric Acid treated, (**e**) Flax_shives biochar_heat_oxid (at 250 °C), (**f**) Beech_ wood biochar_heat_oxid (at 250 °C).

The morphology and structure of the selected biochar before and after modification were investigated using scanning electron microscopy (SEM) and the images are shown in Figure 4. Both the untreated beech and flax biochars showed a high porous structure-rich roughness and irregular surface shape (Figure 4a,b) creating a large volume of irregular rectangular and cylindrical cavities. After the heating treatment at 250 °C or CA treatment, part of the walls of these rectangular channels at the surface of the biochar are destructed and material particles deposit as irregular and granules/cutting on the surface

### 4.2. Surface Morphology Results: BET Surface Area Results

The surface area evaluation applying the BET method (Brunauer-Emmett-Teller) surface area, was carried out for beech and flax biochars before and after CA treatment or heating at 250 °C (Table 3). The beech biochar presented a high surface area of 368 m^2^/g, followed by the flax biochar with 190 m^2^/g. It seems that the brief heating of biochar increases the specific surface of both beech and flax biochar by 5.43 and 70%, respectively. However, the CA treatment leads to a decrease in the biochar surface of the beech and flax biochars with about 60.33% and 22.10% of surface reduction, respectively. Similar results were reported by Singh et al. in the reduction of rice straw-derived biochars treated by poly (diallyldimethylammonium chloride), and it was explained as being caused by the hindered accessibility of the compound on the surface and pores of the material [36]. As shown in Table 3, a variety of SSA values for biochars originating from different biomass feedstocks, production conditions, and post modifications, have been reported in the literature. The biochar reported in the present study lies in the middle-to-high range of these reported values.

It can be observed that the citric acid treatment reduced the available surface area, which at a first glance is not a positive advancement, but the adsorption capacities remained the same or increased compared to the adsorption capacities recorded for the heat-treated samples (samples named -heat_oxid). Therefore, at this point an evaluation of the adsorption results is necessary in order to evaluate the SSA results shown under a different light and to evaluate which treatment improved biochars the most.

Wei Chen et al. tested the MB adsorption on pomelo biochars and showed that the lowest SSA biochar shows comparable adsorption capacity to the other biochars, indicating that the SSA is not the only significant parameter that would positively affect adsorption. However, the biochars tested in this referenced work were not further treated or activated; therefore, no further comparisons can be made. In their work, Dezhi Chen 2017 shows the maximum adsorption capacities of biochars towards CMZ for a large variety of biochars [40], explaining that their tested material shows superior absorption due to the very large SSA but also the pore volume compared to other biochars. No link is made, however, to subsequent functionalization or any trade-offs between surface area increase and surface functionalization.

The flax and beech biochars treated with CA show the lowest SSA but they show the greatest Q_m_ toward methylene blue adsorption. This indicates that the corresponding surface area-normalized maximum adsorption capacities are the greatest and that the surface functionalization by CA enhances the surface properties more than the heat treatment (oxidation treatment). Obviously, the increase in SSA improves the adsorption capacity of biochars and SSA remains an important biochar quality criterion when evaluating materials for wastewater treatment. However, the increased normalized maximum adsorption capacity, which is the ratio of Q_m_ per SSA (mg pollutants per surface area), is the highest despite the reduced surface area of the CA-treated biochars. This leads us to the conclusion that organic acid treatment indeed adds surface functionalities to the biochars, as was the initial assumption on which our study is based.

### 4.3. Thermogravimetric Analysis

**Figure 5:**
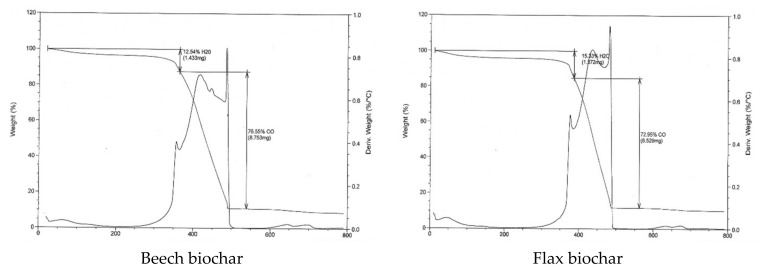

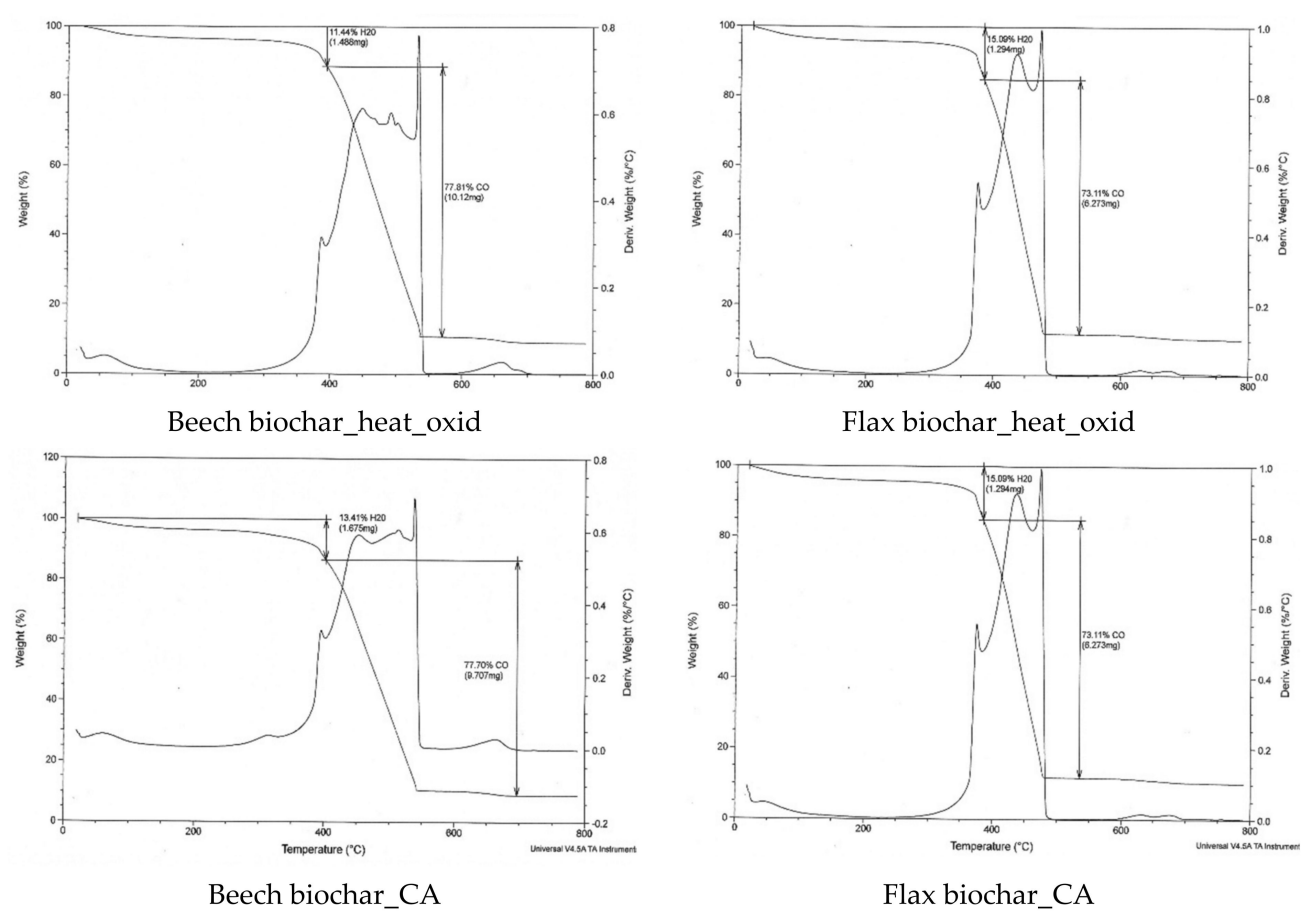
Thermogravimetric analysis of beech and flax biochars before and after modification.

Generally, in thermogravimetric analysis three stages of mass loss take place, first due to the water evaporation (below 250 °C), followed by the thermal decomposition of polar organic compounds derived from cellulose, hemicellulose, and holocellulosic precursors (between 250 and 450 °C), and finally the decomposition of high molecular-weight components between 450 °C and 900 °C [30,36]. As shown in Figure 5, between 11% and 15% of mass loss is observed in the first stage for the different studied biochars with more stability in mass loss for the flax biochar with or without treatment. During the second stage, mass loss is between 73% and 78% for the six analyzed biochars with more polar organic content in the beech biochar samples. The obtained residual mass beyond 800 °C during the third stage (in% *w*/*w*) is estimated at 13, 14, 12, 14, 10, and 2 for beech biochar, flax biochar, beech_heat_oxid, flax_heat_oxid, beech_sCA, and flax_CA, respectively. These results can be explained by the inorganic content more likely present in the biochar residual weight, as reported in Section 4.4, where less residual weight corresponds to a lower total metal content (ICP analysis).

### 4.4. ICP Analysis

ICP-OES analysis was carried out to get an overview of the inorganic elements present in the studied biochars and to explain the results based on certain inorganics present.

Table 4 summarizes the major elements found in the six (06) biochars analyzed, showing a relatively high concentration in Ca and K (concentration from 7.07 to 20.57 mg/g biochar for Ca and from 1.91 to 14.65 mg/g biochar for K) for all biochar samples followed by Fe (from 1.05 to 5.93 mg/g biochar). On the other hand, beech biochar samples, treated or not, contain more elements such as Ni, Mn, and Cr contrary to flax biochar although in relatively low numbers. The results do not imply any significant change caused by the treatments and no trend can be established that can link treatment with a certain degree of elements leaching out, except for the reduction in K in the case of the beech biochar before and after the treatment.

### 4.5. Adsorption Isotherms

The maximum adsorption capacity for beech biochar was evaluated using the Langmuir and Freundlich isotherm models (see Table 5). It seems that the non-linear Langmuir model has the best correlation for the adsorption of both MB and CMZ on natural beech biochar and citric acid-treated beech biochar compared to the Freundlich non-linear model. This indicates its suitability to describe the adsorption, assuming a monolayer coverage and uniform activity distribution on the sorbent surface with a similar activation energy and affinity toward the pollutant. However, the non-linear Freundlich model presented a good correlation for heated beech biochar for MB adsorption only. The Freundlich adsorption isotherm, on the other hand, proposes that adsorption proceeds via a multilayer adsorption formation in which numerous sites with diverse adsorption energies are involved [41].

The non-linear Freundlich model shows the best correlation fit for the adsorption of MB and CMZ on both natural flax biochar and heated flax biochar.

*q_max_* and *K_L_* are the Langmuir constants that are related to the adsorption capacity. The equilibrium data were also fitted to the Freundlich equation. The parameters *K_f_* and *n* estimate the sorption capacity and the sorption intensity of the system. The magnitude of the term (1/*n*) gives an indication of the favorability of the sorbent/adsorbate systems [42]. The model parameters (*K_L_*, *K_f_*, *q_m_*_,_ and *n*) are shown in Table 5.

**Figure 6:**
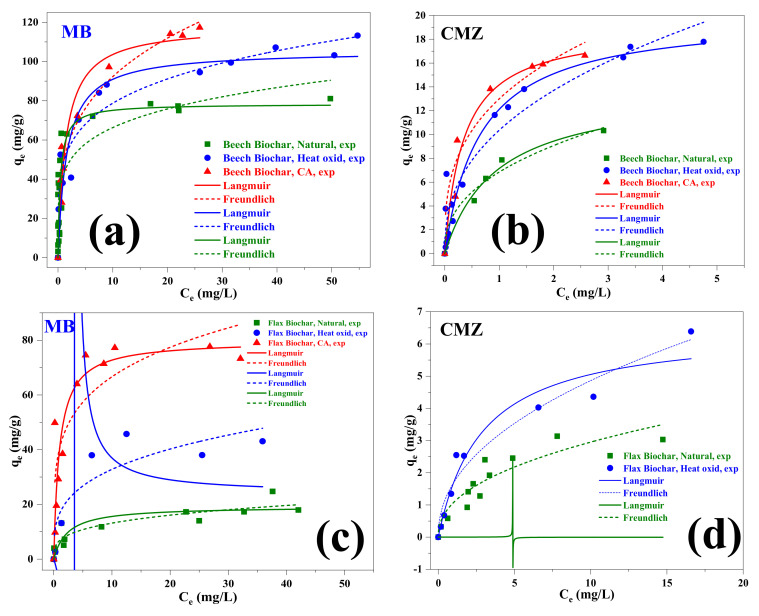
Equilibrium isotherms (smooth lines) and experimental data (points) for: MB adsorption on beech biochar (**a**) and flax biochar (**c**), CMZ adsorption on beech biochar (**b**) and flax biochar (**d**).

The correlation coefficient values of the non-linear Freundlich model were lower than the Langmuir values in most cases. The adsorption equilibrium results for MB and CMZ on the tested biochars in their untreated and treated forms are shown in Figure 6. This figure shows that the Langmuir and Freundlich fits generally have better correlation with the experimentally obtained data. On the same graphs, the experimentally obtained data are shown.

It is also important to note that the obtained experimental adsorption capacity (*q_Exp_*) and Langmuir-calculated adsorption capacity (*q_m_*) presented a good correlation for the major studied biochars that presented a high regression coefficient (R^2^).

Based on the performance of beech wood biochar on methylene blue adsorption, it was decided to further focus on this biochar for further adsorption tests using the carbamazepine molecule as a model pharmaceutical component. Figure 6 summarizes the results of the adsorption of carbamazepine on the beech biochar as well as on treated and untreated flax biochar used as a reference and as a link to the test series with MB. Both citric acid treatment and heating up to 250 °C show a significant improvement in the adsorption capacity compared to the untreated beech biochar or flax biochar, and overall the beech biochar shows a remarkably better absorption capacity compared to the flax biochar. Very often, the adsorption performance of biochars is linked to their specific surface area (SSA). Indeed, the SSA of the beech biochars is higher than the flax biochars’ SSA as reported in Table 3. However, the surface functionality, in this study created by the mild oxidation, is also expected to influence the adsorption performance. An evaluation of the results will be presented in the following paragraphs.

A comparative evaluation is presented and discussed after the results of the SSA are discussed, between the adsorption equilibrium results presented in this study and the results published in the literature. The comparative data shown in Table 3 reveal that the adsorption capacities of beech and flax shives biochars are in line with reported data.

Even though the mild treatments achieved an improvement in the adsorption capacity of the reference materials, overall, the adsorption capacity of our tested biochars remains in the lower performance range compared to the results reported. Our results show that the proposed mild treatments are low-cost and low environmental-impact methods to improve the adsorption capacity of biochars and may complement high-performance biochars or active carbons, therefore reducing the quantities needed for those higher-performing materials. The results of the present study indicate that the studied biochars may be good candidates for agronomic applications, with satisfactory surface areas and a multitude of functional groups.

### 4.6. Functional Groups

**Figure 7:**
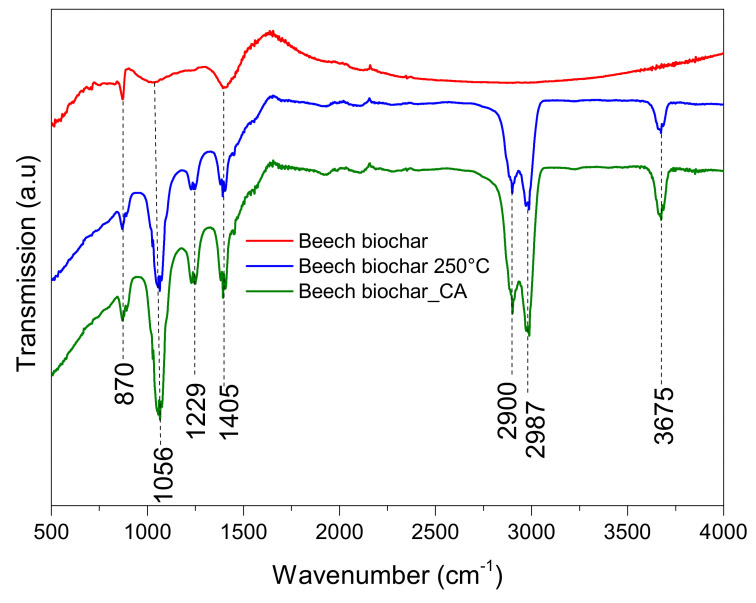
FTIR spectra for beech biochar before and after modification.

**Figure 8:**
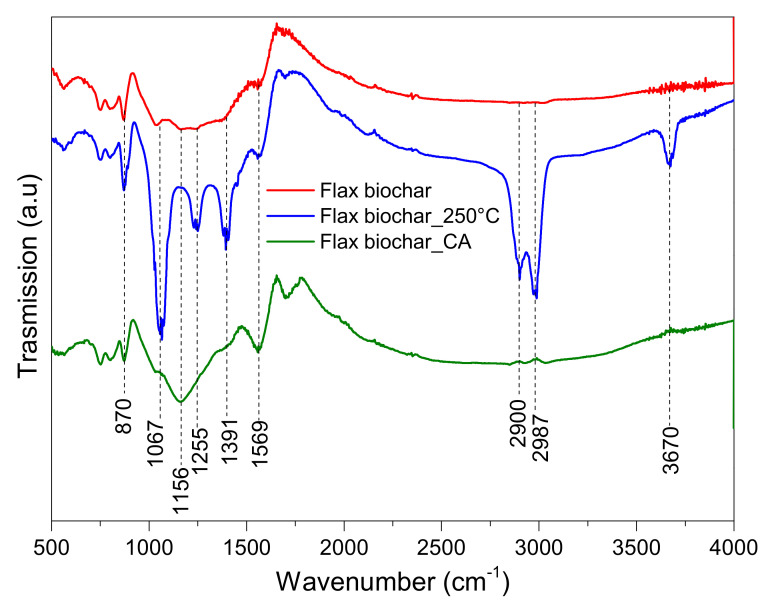
FTIR spectra for flax biochar before and after modification.

FTIR analysis was applied to beech and flax biochars, whether they were without modification, after CA treatment, or after heating at 250 °C to identify characteristic functional groups (Figure 7 and Figure 8). The absorption peak at 3675 cm^−1^ corresponds to the O–H stretching vibration of alcohols, phenols, and carboxylic acids. According to Li et al. and Liang et al. [43,44], this indicates ‘‘free’’ hydroxyl groups on the adsorbent. The peaks at 2987, 1405 or 1391, 1056 or 1067, and 870 cm^−1^ are due to –CH_2_, C=O, C–O, and C–H stretching, respectively [7,45,46]. The heating treatment leads to the appearance of three principal peaks at 2987, 2900, and 1056 (for beech biochar) and 1067 cm^−1^ (for flax biochar), which can be attributed to –CH_2_ and C–O stretching, respectively. Nevertheless, the acidic treatment of beech biochar leads to an increase in both C=O and C–O peak intensities, reflecting the incorporation of a more oxygenated surface group on beech biochar. On the other hand, the CA treatment of flax biochar significantly increases the intensities of C=O and C–O peaks at 1569 and 1156 cm^−1^, respectively [47,48], which can be explained by the fixation of citric acid on the flax biochar surface.

It seems that heating and citric acid treatments have the same impact on surface functional groups of beech biochar while influencing flax biochar differently. It can be seen in Table 3 that the acid treatment reduces the total surface area (SSA) while introducing surface functional groups as shown in Figure 7 and Figure 8. Since the step of acid activation reduced the SSA, the available sites on the biochar surface that could host the added surface functional groups by the citric acid treatment are reduced, which means that the impact of CA functionalization is not added on top of the SSA and therefore does not impose a significant change as expected. The available data do not allow the calculation of the exact tradeoff between SSA and CA treatment benefits and offsets. The explanation remains qualitative. The acid treatment also reduces the flax shives biochar surface area, although it did not have a significant impact on enriching the surface of the material with functional groups.

The functional groups (C–O, C=O, C=C) on the surface of biochar can contribute to the uptake of both classes of dyes (anionic and cationic) via chemical bonding and pi–pi interactions, as shown in the results of Yan Xu et al. [8], who explain that the carboxyl groups on the surface of biochar via the esterification reaction with CA, contribute to the adsorption of MB. Zubai et al. and Chen et al., 2018 propose a three-step adsorption process: (i) rapid film diffusion: dye adsorption associated with chemical interactions comprising electrostatic and chemical binding with oxygen functionalities, (ii) slowdown of intra-particle diffusion of dye molecules into the pores of biochar and (iii) saturation of adsorption sites (equilibrium phase) [7,49]. Based on this proposal the conclusion that is drawn is that surface functional groups contribute to the enhancement of the first phase of the mechanism proposed, allowing the molecules that interact with functional groups to more easily reach the pores available for adsorption.

The mechanism of cationic dyes’ (MB) adsorption process mainly includes pi–pi and chemical interactions with ion exchange. A similar interaction mechanism was also reported by other biochars derived from different biomass for remediation of dye-polluted water bodies [49].

The CMZ molecule is inert in the range of the pH tested; therefore, it may be less dependent on the surface functional groups on the biochar surface, and more dependent on SSA and porosity. Given the fact that flax shives SSA is significantly lower than beech biochar SSA, the CMZ adsorption capacity of flax shives remains insignificant. The poor results on surface area functionalization and the reduction of the SSA of the flax shives biochar discouraged further testing of this material toward CMZ adsorption.

### 4.7. pH and Point of Zero Charge (pHpzc) of the Tested Biochars

The effect of the solution pH on the adsorption of dyes is well-documented, showing that cationic dyes adsorb best under pH values slightly above 6 and preferably above the pH_pzc_ of the substrate. The value of pH = 6 discussed here is justified as follows. MB can be present in an aqueous solution as the cationic species (MB^+^) and undissociated molecules (MB°). At very low pH values, the MB° species predominates (86%, undissociated form) since its pKa is lower than the pH, and the biochar surface is positively charged. Therefore, the adsorption of MB is disfavored by the electrostatic repulsion between the cation MB (the dissociated part of MB) and the surface of the biochar. Both the MB° (50%) and MB^+^ (50%) species coexist at pH values equal to the dissociation factor of MB pH = pKa and for pH values above 6 methylene blue ion is the only species present in the solution [27].

When the solution pH changes, the interactions between the adsorbent surface functionalities and the ionic nature of dye molecules can be affected, influencing the dye’s uptake [7]. It has been concluded that the optimum pH value for the adsorption of MB (cationic dye) is at pH = 6 and slightly above [7,50,51], but any further increase in pH above 8 causes no appreciable increase in the maximum adsorption capacity of the dye. The MB biochar adsorption process might not only be governed by electrostatic interactions but also π–π interactions could be included with the involvement of pore diffusions.

CMZ molecules have a neutral charge within the pH range of our trials because the pKa values of CMZ are 2.3 and 13.9. At values below pH 2.3, CMZ is positively charged, whereas above pH 13.9 it is negatively charged [26]. Therefore, the CMZ molecule not being charged is less affected by pH fluctuations and is surface area-dependent. The conclusion is that the adsorption of CMZ onto biochars may be mostly controlled by hydrophobic and p–p interactions and hydrogen bonding. The reported work in the literature shows a rather positive correlation of carbamazepine adsorption with the solution pH for values between pH = 2 and 6, achieving a maximum adsorption capacity at pH = 6, whereas further increasing of pH was not correlated to a further adsorption capacity increase [40,52].

The sensitivity of the adsorption process as a function of the solution pH was not extensively studied; however, the initial pH was adjusted to the value of pH = 6–6.5, reported as optimum, and the pH values were recorded at the end of the adsorption tests to evaluate the pH fluctuations. In all cases, the end value tended towards the original biochar pH value. The pH values of the biochar used as well as the pH values at the beginning and the end of the tests are shown in Table 6. It seems that the biochars have a strong buffering capacity and regardless of the initial pH solution, at the end of the equilibrium tests the pH tends to reach the pH value of the biochar. Therefore, it is reasonable to conclude that the range of the pH values from the beginning to the end of the tests did not negatively affect the adsorption capacity of the tested biochars.

**Figure 9:**
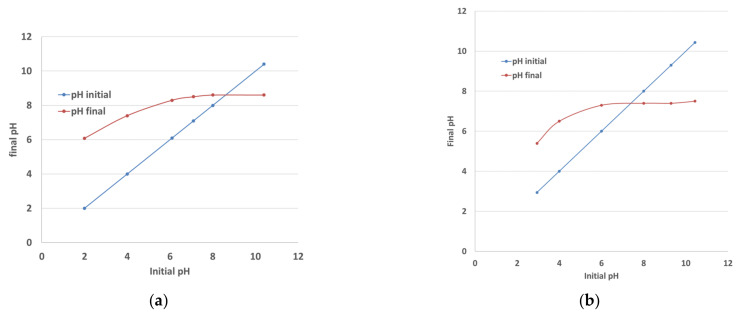
The values of pHpzc of the beech biochar_heat_oxid (**a**) and beech biochar_citric acid treated (**b**).

The pH_pzc_ method was applied to flax and beech biochars. Figure 9 shows an example of the results obtained, i.e., the ‘‘pH drift’’ data per biochar sample from which the pH_pzc_ of the adsorbent can be determined. The pH_pzc_ is the point where the curve pH_final_ vs. pH_initial_ intersects the line pH_initial_ = pH_final_ [22]. The figures show the pH values on both axes. The results of the characterization can be viewed in Table 6. The same table shows the pH values of the biochars by agitating the biochar in demineralized water for 76 h and measuring the pH of the filtered water.

Since the biochar surface is positively charged at pH values < pH_pzc_, the adsorption of MB may have been disfavored at pH values of 6.5, which are the pH values at the beginning of the experiments, because of the electrostatic repulsion between the cation MB and the surface of the biochar. However, in the published literature [29,53,54] we see that the maximum adsorption capacity *q_e_* of MB on a variety of biochars was obtained at pH values between 6 and 8 without further improvement of the adsorption capacity, suggesting that the adsorption process might not only be governed by electrostatic interactions, but also π–π interactions are included with the involvement of pore diffusions. In our tests, the pH value measured during and at the end of the tests was at 8 or above, implying that adsorption of the ionic compounds is favored. Biochar has a strong buffering capacity and regardless of the initial pH solution, at the end of the test after equilibrium is established the pH tends to reach the pH value of the biochar (residual value). Finally, adsorption is largely dependent on the surface area as was commented previously, which assists in the adsorption process regardless of the electrostatic interactions between the biochar and molecules.

In the case of carbamazepine, the pH is not a very significant parameter affecting the adsorption capacity because of the neutral charge of the molecule. Therefore, it appears that the adsorption mechanism of CMZ on biochar is mainly surface area-dependent, which is proven by the very large difference between the adsorption capacity achieved for beech vs. flax shives (large difference in surface area), and the small variations between heat-treated and citric acid-treated beech biochar (small difference in surface area).

### 4.8. Effect of Adsorbent Dose and Initial Dye Concentration

**Figure 10:**
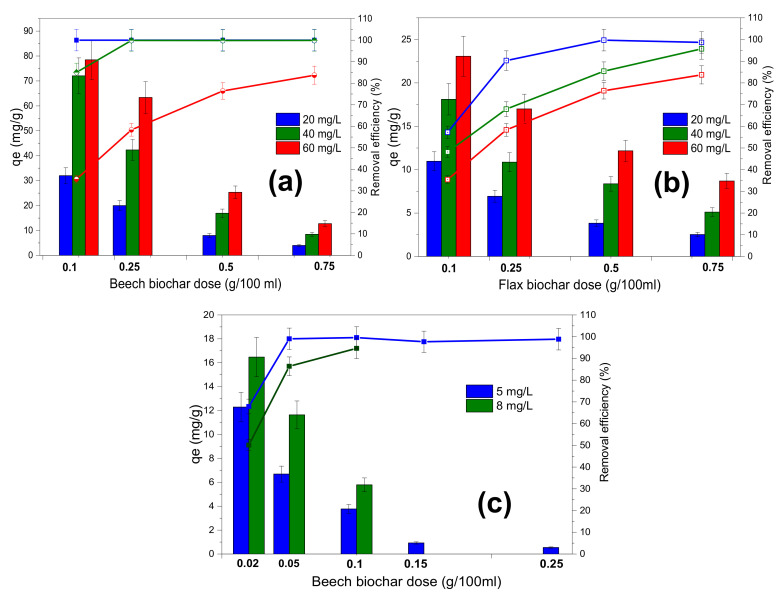
Effect of natural biochar dose on the adsorption capacity (column) and the removal efficiency (line) for: (**a**) beech biochar on MB adsorption, (**b**) flax biochar on MB adsorption, and (**c**) beech biochar on CMZ adsorption.

The equilibrium adsorption capacity *q_e_* and removal efficiency of MB on flax shives and beech biochar is plotted by varying the amount of biochar ranging from 0.1 to 0.75 g/100 mL for an MB concentration range of 20–60 mg/L at 20 °C (Figure 10a,b). In the case of the carbamazepine adsorption trials, only the beech biochar results are shown because the adsorption on flax shives was not significant compared to the beech biochar results (Figure 10c). It should be mentioned that the results presented in this study should be evaluated qualitatively at this stage rather than absolutely. These results reveal a tendency and suggest treatments of biochar that improve their adsorption capacity.

The equilibrium adsorption capacity decreased with an increase in biochar doses (Figure 10) probably due to increased surface area and the availability of more binding sites, while at the same time the concentration of the pollutant molecules was not yet sufficient to saturate these active binding sites [37]. Another reason might be the accumulation and aggregation of biochar particles decreasing the total available surface area and the accessibility of pollutants to the binding sites [37].

The pollutant-removal efficiency increased with the adsorbent dose. This may be due to the availability of more adsorbent sites and, therefore, greater availability of specific surfaces of the adsorbents. The dye-removal efficiency increases as the dye concentrations decrease. This could be due to the increased availability of sites able to bind the pollutants.

The current tests did not reveal a maximum pollutant removal for the range of adsorbent doses and the applied pollutant concentrations.

### 4.9. Desorption Study

Simple trials were carried out by leaving the spent biochar materials in demineralized water for one week and analyzing the water samples for any MB or CMZ, respectively. The objective of these trials was to verify that the pollutants are adequately adsorbed on the biochar in such a way that desorption of the pollutant back to clean water is excluded. In no cases of the samples studied and presented in this manuscript were there any detectable traces of MB or CMZ in the leachates.

## 5. Conclusions and Outlook

On the basis of the results of this evaluation, beech biochar shows satisfactory adsorption capacity for methylene blue and carbamazepine removal. The operating parameters for the maximum sorption of MB were observed for an initial dye concentration of 20 mg/L and a sorbent dosage of 0.05 g/100 mL. The removal of methylene blue and CMZ is enhanced by a thermal treatment that activates the surface and adds oxygen groups or probably reveals or enhances porosity and is mostly enhanced by a citric acid treatment that adds surface functional groups but at the cost of a reduced surface area, as discussed in the study.

Equilibrium data fit well in the Langmuir isotherm models, which confirmed that the sorption is heterogeneous and occurred through physico–chemical interactions, whereas the data did not fit as well in the Freundlich model. Very low-demanding activation steps proved efficient toward a modest-to-satisfactory improvement of the adsorption capacity of the tested biochars. The activation steps include heating the biochars in a standard muffle oven up to 250 °C and citric acid treatment, which seem to have added surface functionalities that increased the sorption capacity of biochars. FTIR data show that the heating of the biochars under air-added oxygen surface groups likely improved the adsorption capacity of the biochar significantly for the low-performing flax biochar. This is an encouraging result towards activating biochars with low environmental-impact methods. The application of mild organic acids for the functionalization of biochars is an economically viable and environmentally friendly method that can contribute to sustainable alternatives to commercial active carbon filters. This work will continue in investigating other organic acids as activating media and heat activation using air and air/oxygen blends for a broad range of well-characterised biochars. The thorough inventory of data on biochar production and activation, adsorption capacity results per pollutant, and in combination with their physicochemical properties will contribute to setting the frame for environmental evaluations to identify trade-offs between the high-efficiency active carbon currently used and lower efficiency biochars locally sourced.

A direct comparison of the studied biochars with active carbon is not straightforward and was not addressed in the study as there exists a variety of products on the market. It is important to keep in mind that the environmental footprint of the various processes together with economic indicators will affect the final decision making towards using one or another product. The environmental impact of various active carbons vs. activated (or functional) biochars is a very important factor to consider when comparing and evaluating processes and should complement a technical study such as the present one. Desorption studies and the reusability potential of the spent material are also important issues that were not addressed in this initial study by our research group. These topics will be addressed in the next planned test trials as we advance our research on biochar modification towards functionalized materials.

## Figures and Tables

**Table 1:** Composition of the beech residues and flax shives (ultimate analysis and main inorganic elements).

Biomass	Ash 550 °C	Vol %	Fixed Carbon%	C %	H %	N %	Cl mg/kg	Ca mg/kg	P mg/kg	K mg/kg	Cu mg/kg	Fe mg/kg
**Flax shives**	1.7	77.6	18.7	48.5	5.8	0.6	380	4149	985	3098	2.8	352
**Beech**	0.9	80.6	17.5	50.3	6.0	0.2	35	1925	270	1150	1.4	348

**Table 2:** Biochars and modified biochars used in the present work.

Biochar	Treatment	Tested Polutants
Beech	Only demineralised water washed (washing)	Methylene blue, Carbamazepine
Beech-heat_oxid	Washing and heating	Methylene blue, Carbamazepine
Beech_CA	Washing and acid treating	Methylene blue, Carbamazepine
Flax shives	Only demineralised water washed (washing)	Methylene blue, Carbamazepine
Flax_heat_oxid	Washing and heating	Methylene blue,
Flax_CA	Washing and acid treating	Methylene blue

**Table 3:** Specific surface area results for the studied biochars and maximum adsorption capacity normalized per surface area.

Adsorbent Material	*q_Exp_* (mg/g)	S_BET_ (m^2^/g)	Q_m_/S_BET_ (mg/m^2^)		Reference
Beech-natural	81.06	368	0.215	Methylene Blue	Present study
Beech-heat_oxid	113.22	388	0.286	Methylene Blue	Present study
Beech_CA	117.33	146	0.835	Methylene Blue	Present study
Flax shives-natural	24.74	190	0.113	Methylene Blue	Present study
Flax-heat_oxid	45.73	272	0.163	Methylene Blue	Present study
Flax CA	77.64	148	0.555	Methylene Blue	Present study
Cattle manure-derived biochar	40	3.6	11.1	Methylene Blue	[37]
Water hyacinth biochar	80	93	0.45	Methylene Blue	[8]
Water hyacinth biochar CA	395	57	6.9	Methylene Blue	[8]
Date Palm Waste biochar B 700 4	206	432	0.478	Methylene Blue	[7]
Date Palm Waste biochar B 800 2	164	268	0.478	Methylene Blue	[7]
Magnolia grandiflora Linn. leaves biochar (MBC).	67	98	0.683	Methylene Blue	[38]
Badam shell biochar (BBC).	25	269	0.09	Methylene Blue	[38]
Pomelo (Citrus grandis) peel biochar (PBC)	32	0.026	-	Methylene Blue	[38]
Beech-natural	10.33	368	0.038	Carbamazepine	Present study
Beech-heat_oxid	17.79	388	0.05	Carbamazepine	Present study
Beech_CA	16.64	146	0.142	Carbamazepine	Present study
Flax shives-natural	3.13	190	0.019	Carbamazepine	Present study
Flax-heat_oxid	6.39	272	0.024	Carbamazepine	Present study
Flax CA	-	148	-	Carbamazepine	Present study
Coconut shell biochar	89.6	365	0.245	Carbamazepine	[39]
Pomelo peel biochar B400	14.75	94	0.157	Carbamazepine	[40]
Pomelo peel biochar AB600	80.64	198.0	0.407	Carbamazepine	[40]
Pomelo peel biochar AB700	286.5	904	0.317	Carbamazepine	[40]
Pinenut and walnut shell biochars	30–45		0.245	Carbamazepine	[39]

**Table 4:** ICP analysis results for different used biochars, before and after treatments.

Concentration (mg/g Biochar)	Ca	Cr	Cu	Fe	K	Mn	Ni	Rb	Zn	Total
**Beech**	20.57	0.42	nn	5.93	14.65	2.63	3.27	nn	nn	47.47
**Beech_** **-heat_oxid**	18.61	0.55	nn	4.69	4.48	2.02	14.69	nn	nn	45.04
**Beech_** **CA**	16.31	<0.30	nn	2.82	2.68	1.96	4.89	nn	nn	28.66
**Flax shives**	13.08	nn	nn	1.05	10.11	<0.25	nn	nn	nn	24.24
**Flax shives_** **-heat_oxid**	12.69	nn	nn	1.99	10.35	<0.25	nn	nn	<0.50	25.03
**Flax shive_** **CA**	7.07	<0.31	nn	<0.31	1.91	<0.26	nn	nn	nn	8.98

nn: non-detected.

**Table 5:** Key values of the Langmuir and Freundlich isotherm plots for the adsorption of MB and carbamazepine onto the tested biochars.

Materials/Isotherms	q_Experimental_ (mg/g)	Langmuir			Freundlich		
		*q_m_* (mg/g)	*K_L_*	R^2^	*K_f_*	*n*	R^2^
MB							
Beech Biochar, Natural	81.06	78.38	2.28	0.68	42.49	0.19	0.68
Beech Biochar, Heat Oxid	113.22	105.6	0.61	0.88	48.45	0.21	0.91
Beech Biochar, CA	117.33	118.23	0.7	0.95	48.61	0.27	0.94
CMZ							
Beech Biochar, Natural	10.33	13.92	1.05	0.98	6.84	0.4	0.91
Beech Biochar, Heat Oxid	17.79	20.05	1.53	0.92	10.36	0.4	0.91
Beech Biochar, CA	16.64	19.05	2.9	0.96	12.99	0.32	0.86
MB							
Flax Biochar, Natural	24.74	19.24	0.42	0.79	6.79	0.28	0.8
Flax Biochar, Heat Oxid	45.73	23.83	−0.28	0.36	16.64	0.29	0.78
Flax Biochar, CA	77.64	79.85	1.01	0.81	40.51	0.21	0.7
CMZ							
Flax Biochar, Natural	3.13	0.00	−0.20	−2.08	1.07	0.43	0.83
Flax Biochar, Heat Oxid	6.39	6.49	0.35	0.94	1.68	0.46	0.95

**Table 6:** Values of pH for the biochars, evolution of the pH value at the beginning and at the end of the experiments, biochar charge 0.1 g/100 mL solution, and values of pH at the point of zero charge.

Sample/Solution	pH	Begin	End	pHpzc
Beech biochar	11.0	-	-	8.2
Beech biochar_heat_oxid	9.9	-	-	8.9
Beech biochar, CA	9.9	-	-	7.3
Solution MB 20–50 mg/L, with beech		6.5	8.3–8.7	
Solution MB 50–100 mg/L, with beech		6.0	8.1–8.5	
Solution CMZ 1–20 mg/L, with beech		8.4	8.5–9.0	
Flax biochar	10.4	-	-	8.0
Flax biochar_heat_oxid	10.5	-	-	8.1
Flax biochar, CA	7.8	-	-	7.1
Solution MB 20–50 mg/L, with flax		6.5	7.7–7.9	
Solution MB 50–100 mg/L, with flax		6.0	7.5	
Solution CMZ 1–20 mg/L, with flax		7.7	7.9	

## Data Availability

The datasets generated during and/or analyzed during the current study are not publicly available due to confidentiality characters of the ThreeC project but are available from the corresponding author on reasonable request.

## Supplementary Materials

The following supporting information can be downloaded at: https://www.mdpi.com/article/10.3390/ma15082824/s1, Figure S1: Calibration curve of (a) methylene blue dye and (b) carbamazepine.
