# Supplementary material for: Potential of Flax Shives and Beech Wood-Derived Biochar in Methylene Blue and Carbamazepine Removal from Aqueous Solutions
Source: Materials (Basel). 2022 Apr 12;15(8):2824. doi: 10.3390/ma15082824 (PMC9029730; doi:10.3390/ma15082824)
Supplement: Supplementary file 1 [file materials-15-02824-s001.zip › materials-1632433-supplementary.pdf]

Supplementary information

Potential of Flax shives and Beech wood derived biochar in methylene blue and carbamazepine removal from aqueous solutions

Hicham ZEGHIOUD[1*], Lydia FRYDA[1], Angélique MAHIEU[1], Rian VISSER[2], Abdoulaye KANE[1]

[1] UniLaSalle - Ecole des Métiers de l'Environnement, Research unit CYCLANN, Campus de Ker Lann, 35170 Bruz, France; lydia.fryda@unilasalle.fr; angelique.mahieu@unilasalle.fr; abdoulaye.kane@unilasalle.fr
[2] TNO Dutch Institute of applied research, Department of Energy Transition, Westerduinweg 3, Petten. Postal Code, 1755 LE. Netherlands; rian.visser@tno.nl
* Correspondence: hicham.zeghioud@unilasalle.fr

The calibration of the spectra for both methylene blue (MB) and carbamazepine (CMZ) as shown in Figure S1 is valid for the pH range of 6 – 10. Above these values the absorbance increases for the same concentration and the calibration needs to be verified.

a) **b)**

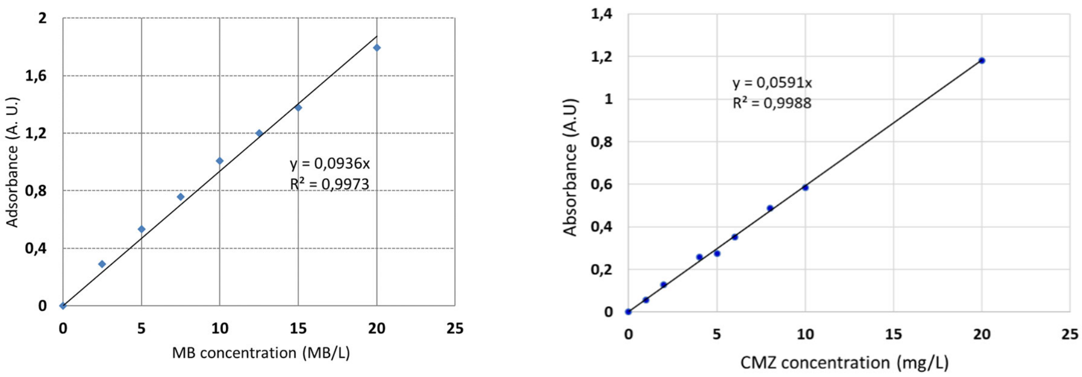

Figure S1. Calibration curve of a) methylene blue dye and b) carbamazepine.
